# Reasons for non-recruitment of eligible patients to a randomised controlled trial of secondary prevention after intracerebral haemorrhage: observational study

**DOI:** 10.1186/s13063-017-1909-4

**Published:** 2017-04-05

**Authors:** Amy E. Maxwell, Mary Joan MacLeod, Anu Joyson, Sharon Johnson, Hawraman Ramadan, Ruth Bellfield, Anthony Byrne, Caroline McGhee, Anthony Rudd, Fiona Price, Evangelos Vasileiadis, Melinda Holden, Jonathan Hewitt, Michael Carpenter, Ann Needle, Stacey Valentine, Farzana Patel, Frances Harrington, Paul Mudd, Hedley Emsley, Bindu Gregary, Ingrid Kane, Keith Muir, Divya Tiwari, Peter Owusu-Agyei, Natalie Temple, Lakshmanan Sekaran, Suzanne Ragab, Timothy England, Amanda Hedstrom, Phil Jones, Sarah Jones, Mandy Doherty, Mark O. McCarron, David L. Cohen, Sharon Tysoe, Rustam Al-Shahi Salman

**Affiliations:** 1 Centre for Clinical Brain Sciences, University of Edinburgh, Chancellor’s Building, 49 Little France Crescent, Edinburgh, EH16 4SB UK; 2 Aberdeen Royal Infirmary, Aberdeen, UK; 3 Barnsley Hospital NHS Foundation Trust, Barnsley, UK; 4 Bradford Royal Infirmary, Bradford, UK; 5 Forth Valley Royal Hospital, Larbert, UK; 6 Guys & St Thomas, London, UK; 7 Hereford County Hospital, Hereford, UK; 8 Hillingdon Hospital, Uxbridge, UK; 9 Ystrad Mynach Hospital, Newport, UK; 10 Pinderfields Hospital, Wakefield, UK; 11 Queen Alexandra Hospital, Portsmouth, UK; 12 Royal Blackburn Hospital, Blackburn, UK; 13 Royal Cornwall Hospital, Cornwall, UK; 14 Royal Devon & Exeter Hospital, Exeter, UK; 15 Royal Preston Hospital, Preston, UK; 16 Royal Sussex County Hospital, Brighton, UK; 17 South Glasgow University Hospital, Glasgow, UK; 18 The Royal Bournemouth Hospital, Bournemouth, UK; 19 Peterborough City Hospital, Peterborough, UK; 20 Luton & Dunstable NHSFT University Hospital, Luton, UK; 21 Poole Hospital, Poole, UK; 22 Derby Royal Hospital, Derby, UK; 23 Bronglais Hospital, Aberystwyth, UK; 24 South West Acute Hospital, Enniskillen, UK; 25 Altnagelvin Hospital, Londonderry, UK; 26 Northwick Park Hospital, Harrow, UK; 27 Southend University Hospital NHS Foundation Trust, Southend-on-Sea, UK

**Keywords:** Stroke, Intracerebral haemorrhage, Recruitment, Screening log

## Abstract

**Background:**

Recruitment to randomised prevention trials is challenging, not least for intracerebral haemorrhage (ICH) associated with antithrombotic drug use. We investigated reasons for not recruiting apparently eligible patients at hospital sites that keep screening logs in the ongoing REstart or STop Antithrombotics Randomised Trial (RESTART), which seeks to determine whether to start antiplatelet drugs after ICH.

**Method:**

By the end of May 2015, 158 participants had been recruited at 108 active sites in RESTART. The trial coordinating centre invited all sites that kept screening logs to submit screening log data, followed by one reminder. We checked the integrity of data, focused on the completeness of data about potentially eligible patients and categorised the reasons they were not randomised.

**Results:**

Of 108 active sites, 39 (36%) provided usable screening log data over a median of ten (interquartile range = 5–13) months of recruitment per site. During this time, sites screened 633 potentially eligible patients and randomised 53 (8%) of them. The main reasons why 580 patients were not randomised were: 43 (7%) patients started anticoagulation, 51 (9%) patients declined, 148 (26%) patients’ stroke physicians were not uncertain about using antiplatelet drugs, 162 (28%) patients were too unwell and 176 (30%) patients were not randomised due to other reasons.

**Conclusion:**

RESTART recruited ~8% of eligible patients. If more physicians were uncertain about the therapeutic dilemma that RESTART is addressing, RESTART could have recruited up to four times as many participants. The trial coordinating centre continues to engage with physicians about their uncertainty.

**Trial registration:**

EU Clinical Trials, EudraCT 2012-003190-26. Registered on 3 July 2012.

## Background

Research into methods to boost recruitment has been identified as the highest priority for randomised controlled trials (RCTs) methodological research in the UK [1]. *The Lancet*’s series on ‘Research: Increasing Value and Reducing Waste’ identified under-recruitment to RCTs as a major source of inefficiency in the conduct of applied clinical research [2]. Slow recruitment is particularly inefficient because it delays the delivery of research and inflates its costs by increasing the number of staff and sites or by extending the amount and duration of funding required.

This problem has not been small – a review of 114 RCTs funded by MRC or HTA in the UK in 1994–2002 found that less than one-third recruited their original target within the time originally specified and around one-third were given extensions to achieve their target [3]. A re-investigation of this in 2002–2008 found that almost half of the RCTs did not recruit their originally specified target sample size and nearly half of the RCTs received an extension of some kind [4], which was only a marginal improvement. Recruitment is jeopardised by many factors including restrictive eligibility criteria and inefficient methods for approaching potential participants [5, 6]. Screening logs may help inform the representativeness of a RCT [7], but they may also be informative for understanding why eligible patients are not recruited [8, 9].

Stroke due to intracerebral haemorrhage (ICH) affects ~10,000 adults in the UK each year. Within one month of ICH ~40% of patients die and more than half the survivors are dependent [10]. Achieving large sample sizes in ICH RCTs is therefore challenging, especially when protocols specify short time windows for recruitment after ICH onset and include many eligibility criteria [5]. Many ICH RCTs have studied acute and rehabilitation interventions, but only one published RCT has investigated an intervention for secondary prevention; PROGRESS recruited 611 patients with ICH at 172 hospital sites in ten countries over 30 months in 1995–1997 [11, 12].

The REstart or STop Antithrombotics Randomised Trial (RESTART, ISRCTN71907627 www.restarttrial.org) is an ongoing RCT comparing policies of restarting versus avoiding antiplatelet drugs for secondary prevention after ICH. RESTART aimed to recruit 720 participants over two years (from May 2013 to May 2015) based on recent epidemiological data [5, 13]. RESTART has implemented as many as possible of the strategies that have been shown to maximise recruitment [5, 14, 15] including: minimising the number of eligibility criteria, maximising the time window for recruiting patients after ICH onset, and having an open design where participants know which treatment they are receiving. In addition, the Chief Investigator has used qualitative methods with Principal Investigators at all active sites to identify and overcome barriers to clinician recruitment activity, as recommended by a systematic review [16].

Based on pilot data in an international collaboration [17], 20% of all ICH patients (2% of all strokes) should be potentially eligible for RESTART (although these data were unable to take account of patients’ functional status and a recent hospital-based study found that only 15% of patients form the Lund Stroke Registry were potentially eligible for RESTART [18]). We completed a quantitative investigation of recruitment estimates given by sites when being activated to RESTART in February 2015, and at the time of this investigation 101 sites expected to have recruited 741 patients, but they had in fact recruited 135 (18%). By the end of May 2015, 108 hospital sites were active and 158 participants had been randomised in RESTART (22% of the recruitment target of 720 participants).

Therefore, we sought to identify the main reasons for fewer participants being recruited than expected.

## Methods

To investigate why potentially eligible patients were not being randomised in RESTART we requested sites provide us anonymised screening logs. Although sites had not been asked to specifically keep screening logs for the trial, the trial coordinating centre were aware that some centres kept them for their stroke patients as that was their practice or they were required to by their R&D department. By gathering these valuable data, we could analyse what the leading reasons for not recruiting potentially eligible patients were.

The RESTART trial coordinating centre invited all sites by email on 7 May 2015, asking them if they could provide anonymised logs (if they kept them) containing a list of their patients with ICH who had been screened at their hospital site since becoming active in RESTART along with the outcome e.g. ‘randomised’, ‘not eligible’, ‘patient declined’, ‘doctor not uncertain’, etc. We sent a reminder email on 22 May 2015 to maximise responses by the deadline of 31 May 2015.

The RESTART eligibility criteria are as follows. Inclusion criteria: patient age ≥ 18 years; spontaneous primary or secondary ICH; patient had taken antithrombotic drug(s) for the prevention of vaso-occlusive disease before ICH onset; randomisation more than 24 h after ICH onset; patient and their doctor are uncertain about whether to start or avoid antiplatelet drugs; patient is registered with a general practitioner; brain imaging that first diagnosed the ICH is available; participant or representative consent. Exclusion criteria: ICH due to preceding trauma or haemorrhagic transformation of ischaemic stroke; patient is taking an anticoagulant drug following ICH; patient is pregnant, breastfeeding or of childbearing age and not taking contraception; patient and carer unable to understand spoken or written English.

We asked RESTART hospital sites with available screening log data to record: the date the patient was screened; whether the patient’s diagnosis of ICH was within the two months before screening; whether the patient was screened as an inpatient or outpatient; and the outcome of the screening (ineligible, eligible and randomised, or eligible but not randomised). We categorised reasons for non-recruitment of potentially eligible patients (according to the eligibility criteria above) as follows, and assumed that investigators had recorded the leading reason if there had been more than one:

- Patient declined
- Doctor not uncertain
- Started anticoagulant
- Unwell / end of life / died
- Other

We reviewed the data integrity, completeness and consistency of each submission of screening log data. We collated data in an Excel spreadsheet and recorded key information from each site’s submission, including: the time period of screening log data collection, the number of patients screened, the number of patients that were randomised to RESTART, whether patients were ineligible and, if eligible, the reasons why they were not recruited (categorised as above).

## Results

Of the 108 active RESTART sites emailed to invite them to provide their screening logs, 44 (41%) provided data, but we were unable to use data provided by five sites, leaving data from 39 (36%) of RESTART hospital sites.

The 39 sites that returned screening logs recruited 58 patients between May 2013 and May 2015 (1.49 patients per site) and the 69 sites that did not provide screening logs recruited 100 patients during the same (1.45 patients per site), providing no evidence of bias among the sites included in our analyses of screening logs.

**Fig. 1:**
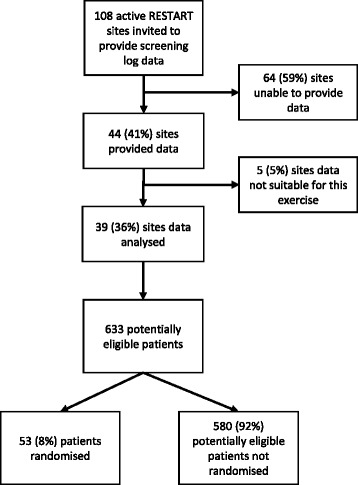
*Flowchart* of the identification and analysis of pertinent screening log information

The data from the 39 sites with screening logs covered a median of ten (interquartile range = 5–13) months per site. During this time, sites randomised 53 participants and also screened 580 patients who appeared eligible but were not randomised (ratio 1:11) (Fig. 1).

**Fig. 2:**
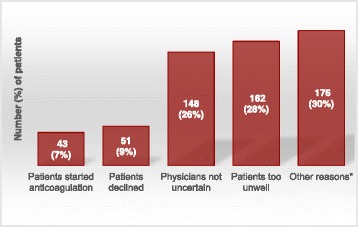
*Graph* showing reasons for 580 potentially eligible patients not being randomised

The main reasons why the 580 patients were not randomised were: 43 (7%) patients started anticoagulation; 51 (9%) patients declined; 148 (26%) had stroke physicians who were not uncertain about using antiplatelet drugs; 162 (28%) patients were too unwell; and 176 (30%) patients were not randomised due to other reasons, for example, lived out of catchment area, transferred to a different hospital, lost to follow-up, enrolled in another drug trial, still under review, consented but not recruited or no clear reason was provided (Fig. 2).

## Discussion

Screening log data demonstrate that 8% of potentially eligible patients were recruited and randomised to the RESTART trial of secondary prevention after stroke due to ICH. The two leading single reasons for eligible patients not being recruited was that they were too unwell/disabled and that physicians were not uncertain despite the clear shortage of evidence to inform this therapeutic dilemma in the available literature [19, 20].

This study has some strengths. The RESTART trial reflects contemporary clinical practice and trial recruitment at a large number of hospitals in the UK. RESTART is the only ongoing RCT of secondary prevention after ICH in the UK. We gave all sites the opportunity to participate, checked the data that we received and used a standardised approach to categorising reasons for non-recruitment.

This study has some weaknesses. RESTART did not mandate sites to keep screening logs – so they were only available at a minority of sites – but used those that were available because sites recorded screening activity to monitor their research activity. Sites returned data in a variety of formats and collected data over slightly differing time periods because there was variation in the time at which they became active in the collaboration. We had to omit some data due to their quality or completeness.

Screening logs appear useful for identifying the leading reasons why eligible patients are not recruited to a RCT. These data can inform future RCTs of pharmacological secondary prevention RCTs after ICH, in which ~8% of eligible participants may be recruited, although we cannot exclude the possibility that this proportion is intervention-specific. Physician certainty, despite the lack of rigorous evidence to inform therapeutic decisions, remains one of the major, potentially modifiable barriers to recruiting larger proportions of eligible patients.

Due to under-recruitment, RESTART’s funder kindly granted an extension of recruitment until 31 May 2018.

## Conclusion

Screening log data can be useful for identifying reasons for low recruitment of eligible patients to a RCT. Approximately 8% of eligible patients were recruited to the RESTART trial. During the period of this screening log analysis, if some physicians had not been certain about whether to start antiplatelet drugs after ICH for 148 potentially eligible patients, RESTART could have almost quadrupled the number of participants recruited from 53 to 201.

## Abbreviations

CI: Chief Investigator
ICH: Intracerebral haemorrhage
RCT: Randomised controlled trial
RESTART: REstart or STop Antithrombotics Randomised Trial
